# Supplementary material for: Cloud BioLinux: pre-configured and on-demand bioinformatics computing for the genomics community
Source: BMC Bioinformatics. 2012 Mar 19;13:42. doi: 10.1186/1471-2105-13-42 (PMC3372431; doi:10.1186/1471-2105-13-42)
Supplement: Additional file 1 — Supplementary 1 Cloud BioLinux software documentation in the form of a mini, self-contained website. Users need to download and uncompress the .zip file, and open through a web browser the "index.html" file available on the main directory. (ZIP 1823 kb). [file 1471-2105-13-42-S1.ZIP › Cloud-BioLinux-Package-Documentation/docs/spin.html]

Bio-Linux Software Documentation Pages

Back to search form

## spin

|  |  |
| --- | --- |
| Name | spin |
| Description |

**Spin** can compare nucleic acid against nucleic acid, protein against protein, and protein against nucleic acid. It can analyse nucleotide sequences to find genes, restriction sites, motifs, etc, as well as performing translations, finding open reading frames, counting codons, etc.

See also information about **pregap4**, **gap4** and **trev**.

  
  |
| Homepage | http://sourceforge.net/projects/staden/ |
| Remote Documentation | http://staden.sourceforge.net/ |
